# Spectroscopic and Chemometric Evaluation of the Stability of Timolol, Naphazoline, and Diflunisal in the Presence of Reactive Excipients Under Forced Degradation Conditions

**DOI:** 10.3390/molecules30183807

**Published:** 2025-09-19

**Authors:** Anna Gumieniczek, Marek Wesolowski, Anna Berecka-Rycerz, Edyta Leyk

**Affiliations:** 1 Department of Medicinal Chemistry, Faculty of Pharmacy, Medical University of Lublin, Jaczewskiego 4, 20-090 Lublin, Poland; anna.berecka-rycerz@umlub.edu.pl; 2 Department of Analytical Chemistry, Faculty of Pharmacy, Medical University of Gdansk, Gen. J. Hallera 107, 80-416 Gdansk, Poland; marek.wesolowski@gumed.edu.pl (M.W.); edyta.leyk@gumed.edu.pl (E.L.)

**Keywords:** timolol, naphazoline, diflunisal, excipients, forced degradation, high temperature/high humidity, UV/Vis light, FT-IR/ATR spectroscopy, NIR spectroscopy, chemometrics

## Abstract

It was previously demonstrated that timolol (TIM), naphazoline (NAPH), and diflunisal (DIF) are susceptible to degradation when exposed to extreme pH conditions and UV/Vis light. However, their stability in the presence of pharmaceutical excipients remains largely unexplored. Thus, their binary mixtures (1:1 ratio, *w*/*w*) with five excipients, hydroxyethyl cellulose (HCA), mannitol (MAN), poly(vinyl alcohol) (PVA), poly(vinylpyrrolidone) (PVP), and Tris HCl (TRIS), were subjected to forced degradation (70 °C/80% RH and UV/Vis light in the dose 94.510 kJ/m^2^). Forced degradation was designed to accelerate potential interactions between these compounds, allowing the earlier identification of degradation risk compared to formal stability studies. FT-IR/ATR and NIR spectroscopy, along with chemometric evaluation using Principal Component Analysis (PCA) and Hierarchical Cluster Analysis (HCA), was applied to assess changes in the spectra, compared to individual compounds and the non-stressed mixtures. A hybrid approach, combining visual assessment with chemometric evaluation of the spectral data, enabled the detection of changes that were not clearly observable using a single analytical method. In particular, interactions of TIM, NAPH, and DIF with MAN and TRIS were clearly identified, while the mixtures of NAPH with excipients proved to be the least sensitive to forced degradation.

## 1. Introduction

The drugs examined in the present study, i.e., timolol (TIM), naphazoline (NAPH), and diflunisal (DIF), are relatively small synthetic molecules (Figure S1, Supplementary Materials) with distinct physicochemical and pharmacological properties. These compounds could be administered in various topical forms, such as eye or nasal drops, skin-applied emulsions, or ointments, creams, and gels, as well as in oral tablets or capsules. TIM is a non-selective β-blocker primarily prescribed in the form of eye drops to lower intraocular pressure in patients with open-angle glaucoma and ocular hypertension. Oral timolol may also be included in poly-therapies of hypertension, myocardial infarction, and migraine prophylaxis [1]. NAPH is a fast-acting sympathomimetic vasoconstrictor that targets the arterioles of the eye and nasal passages. It is commonly used in eye and nasal preparations to relieve congestion associated with severe rhinitis caused by colds, allergic reactions, or inflammatory conditions [2]. DIF is a non-steroidal anti-inflammatory drug (NSAID) with pharmacologic actions similar to other agents in this class, particularly salicylic acid derivatives. It is primarily administered orally to alleviate pain accompanied by inflammation and for the treatment of rheumatoid arthritis or osteoarthritis. Additionally, DIF has been investigated for use in topical formulations and transdermal systems, such as ointments, creams, gels, nanoparticles, and nanoemulsions [3].

It is well established that many excipients used in the drug technology can interact with active pharmaceutical ingredients (APIs), potentially accelerating their degradation or resulting in the formation of specific degradation products. Such incompatibilities of active substances and excipients are possible due to reactive functional groups present in both and can be accelerated under high temperature, humidity, light, or oxygen [4,5,6]. While optimal conditions of temperature, humidity, and light are recommended for formal stability studies of individual APIs, as well as during the manufacturing, transportation, and storage of final formulations, routine stability testing is typically conducted under conditions representative of the target climatic zone (long-term testing). To expedite the assessment, accelerated stability tests are also performed under moderately severe conditions. However, additional testing under more extreme conditions, referred to as stress conditions or forced degradation, is also recommended by regulatory authorities [7]. The primary purpose of forced degradation is to increase the likelihood of detecting potential interactions between drugs and excipients by accelerating the rate of any reactions that may occur between them [8]. These tests provide valuable insights for formulation design and manufacturing by identifying excipients that may compromise API stability, thereby supporting science-driven decisions and minimizing the risk of instability in the final product [9].

Several studies from the literature indicated the sensitivity of TIM, NAPH, and DIF to external conditions, particularly to strong acidic, basic, oxidative, and photolytic conditions. Recent publications focusing on TIM [10], NAPH [11], and DIF [12] present robust examples of forced degradation studies that enable quantitative analysis of these APIs in the presence of their degradation products. Additionally, a number of studies specifically examining the effects of light exposure on the stability of TIM, NAPH, and DIF were published [13,14,15,16,17,18]. However, investigations into the influence of various excipients on the stability of these APIs, especially under stressed conditions, are notably limited. This represents a significant gap, given the potential for excipient–API interactions to impact drug stability and product performance.

Hydroxyethyl cellulose (HEC) is extensively used in the drug technology due to its nontoxic profile and favorable rheological and mechanical properties. For instance, it has been used to develop prodrugs of several NSAIDs to enhance their dissolution and bioavailability and reduce gastrointestinal toxicity [19,20]. However, it is synthesized from cellulose and ethylene oxide, so residues can include unreacted ethylene oxide, affecting drug stability [21]. Mannitol (MAN) was also reported to enhance bioavailability of various APIs by creating pores within the dosage form matrix, thereby facilitating their release [22]. On the other hand, there are reports indicating potential adverse effects of MAN on drug stability, mainly due to the presence of trace amounts of reducing sugars [23]. Poly(vinyl alcohol) (PVA) is commonly produced by hydrolyzing polyvinyl acetate, a process that can result in the presence of residual polyvinyl acetate, acetic acid, and methyl acetate in the final polymer. These impurities have the potential to interact with amino and hydroxyl groups in various APIs, potentially leading to the formation of specific products [24,25,26]. Poly(vinylpyrrolidone) (PVP) was found to be incompatible with a broad range of APIs, particularly in the presence of water [24]. This incompatibility is mainly attributed to the carbonyl groups in PVP, which can form hydrogen bonds with other polar compounds [27,28]. In recent years, there has been a growing interest in Tris (TRIS) as a promising pharmaceutical excipient. It could be used as an excellent buffering agent or to produce water-soluble salts in the case of poorly soluble drugs. For example, TRIS was found to improve the dissolution of some NSAIDs like ketoprofen [29] and nimesulide [30].

In the study of Merino-Bohórquez et al., the stability of TIM in a gel formulation containing poly(acrylic acid) was studied after storage at 25 °C [31]. In another study, various polymers including methyl cellulose, hydroxypropyl cellulose, ethyl cellulose, and PVP were incorporated into ocular inserts with TIM and subjected to accelerated degradation [32]. Additionally, the physicochemical properties of new ophthalmic formulations containing TIM with different polymers, including HEC, were investigated [9]. Regarding NAPH, Shetty and Rompicherla assessed the stability of formulations containing poly(acrylic acid) and hydroxypropyl methylcellulose after storage at 40 °C [33]. In the case of DIF, compatibility studies were conducted with various solid lipids like glycerol dibehenate, cetyl palmitate, stearic acid, glyceryl monostearate, and hydrogenated castor oil [34]. Moreover, coprecipitates of DIF with PVP, as well as their physical mixtures in different drug–excipient ratios, were also examined [35].

Considering the widespread use of TIM, NAPH, and DIF in various pharmaceutical formulations, along with their distinct chemical properties and potential reactivity, the primary goal of this study was to investigate their stability in the presence of five potentially reactive excipients, under forced degradation conditions. Forced degradation was selected as a rapid and informative approach to better understand drug and drug product stability within a shortened timeframe [36]. The excipients were chosen based on their diverse chemical structures (Figure S1, Supplementary Materials) and properties, as well as functional roles in pharmaceutical formulations. As described above, each of these excipients has been reported in the literature to exhibit potential incompatibilities with various APIs, with the interactions likely attributable to reactive functional groups or residual synthesis by-products. Their roles in the design of potential new formulations containing TIM, NAPH, and DIF or APIs similar to them, for example in the forms of enhanced dissolution, were taken into account. The lack of studies addressing these specific drug–excipient interactions further justified this selection.

The second goal of the present study was to evaluate the utility of two spectroscopic methods such as FT-IR/ATR and NIR, combined with chemometric methods like Principal Component Analysis (PCA) and Hierarchical Cluster Analysis (HCA), to detect possible interactions between the studied APIs and excipients. This approach proposes the use of relatively simple, cost-effective, and non-destructive analytical methods to assess drug–excipient compatibility, particularly during the early stages of pharmaceutical product development.

## 2. Results and Discussion

### 2.1. FT-IR/ATR and NIR Analysis

#### 2.1.1. Results for Individual APIs and Individual Excipients

The FT-IR/ATR and NIR spectral characteristics of all individual drugs excipients are summarized in Table S1 (Supplementary Materials). The results indicated no visible changes in the FT-IR/ATR and NIR spectra of substances tested, both APIs and excipients, after exposure to high temperature/high humidity or UV/Vis light, suggesting their stability under the applied stress conditions. The only exception was observed in the FT-IR/ATR spectrum of the light-stressed TIM, where notable changes were detected, as described below. The FT-IR/ATR spectra (excluding TIM) and NIR spectra of all individual APIs and excipients are presented in Figures S2–S4 (Supplementary Materials).

**Figure 1:**
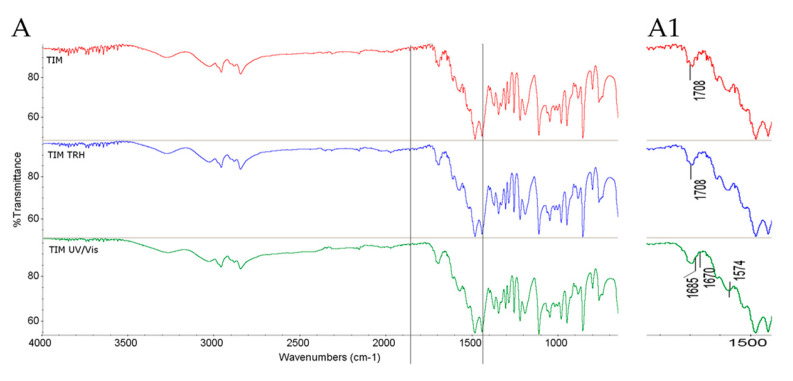
FT-IR/ATR spectra of TIM (**A**,**A1**) in non-stressed and stressed samples where (**A1**) denotes the zoomed parts of the spectra; TRH = the sample stressed with high temperature and high humidity; UV/Vis = the sample stressed with UV/Vis light.

Interestingly, individual TIM exposed to UV/Vis light exhibited distinct changes in its FT-IR/ATR spectrum; specifically, the band at 1708 cm^−1^ attributed to the C=O group in the maleate salt was observed to either disappear or overlap with adjacent bands at 1685 and 1670 cm^−1^, corresponding to C–N vibrations (Figure 1A,A1). In the literature, there are no similar papers concerning the stability of TIM. However, in the paper of Wang et al., enalapril maleate was used for hot-melt extrusion, and some changes concerning the C=O group of the maleate were reported. It was postulated that one carboxylic group of the maleate could interact with its other carboxylic group, or with the amino group of the drug [37]. Similar observations were reported in the literature, where some changes concerning the C=C bond in enalapril maleate moiety were observed and the possibility of photodegradation via photoisomerization to more stable fumarate was suggested [38].

#### 2.1.2. Results for Binary Mixtures of TIM

**Figure 2:**
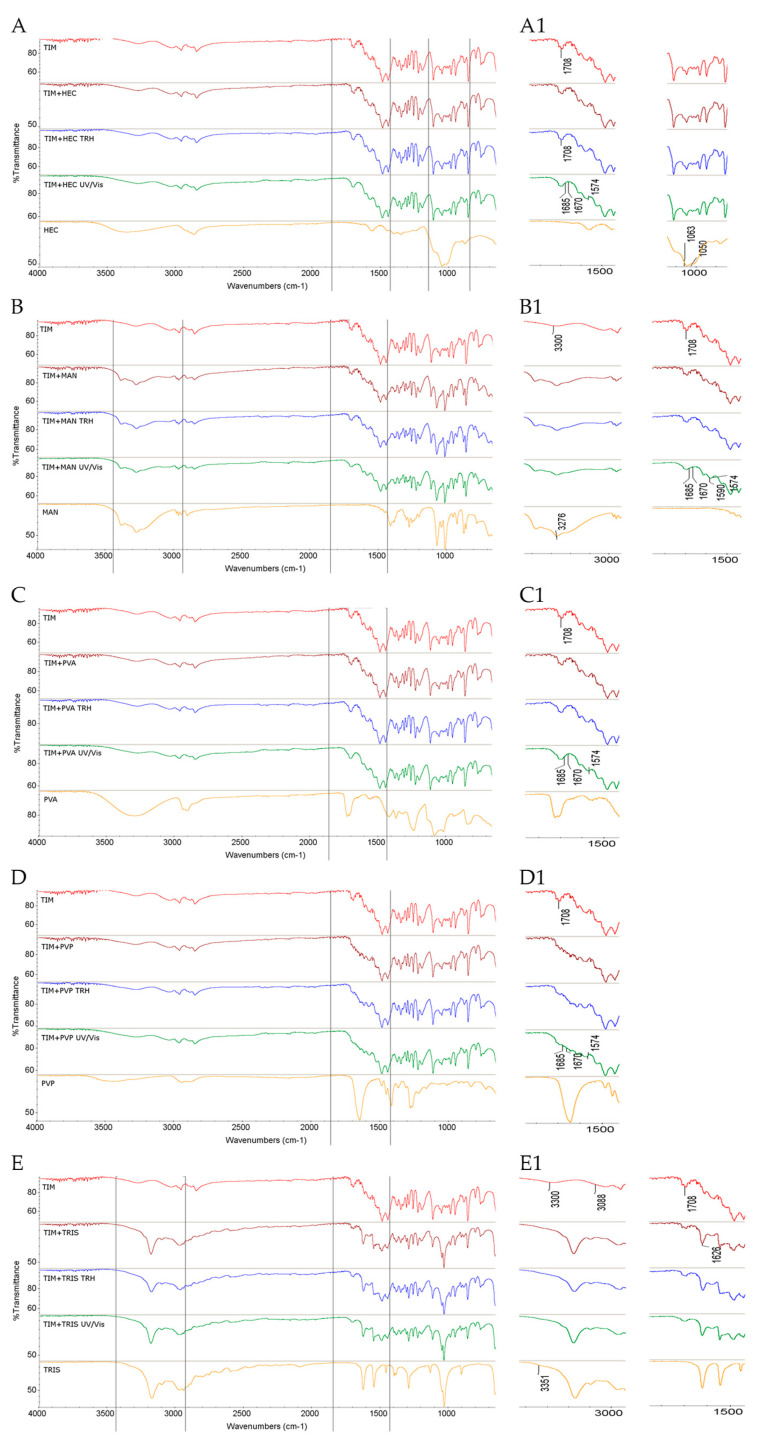
FT-IR/ATR spectra of TIM in binary mixtures with the following: (**A**,**A1**) HEC, (**B**,**B1**) MAN, (**C**,**C1**) PVA, (**D**,**D1**) PVP, and (**E**,**E1**) TRIS in the non-stressed and stressed samples where (**A1**) and onward denote the zoomed parts of the spectra; TRH = the sample stressed with high temperature and high humidity, UV/Vis = the sample stressed with UV/Vis light.

The FT-IR/ATR spectrum of the non-stressed mixture of TIM with HEC showed no significant changes in the absorption pattern of TIM, indicating no detectable interactions under ambient conditions. In previous studies, ophthalmic formulations containing TIM and various polymers, including HEC, were examined, showing the distinct spectral changes in the corresponding FT-IR spectra and suggesting potential interactions, although the stability of TIM under formal stability conditions was reported as satisfactory [9]. In the present study, TIM–HEC mixtures exposed to high temperature and high humidity also did not exhibit visible spectral changes. This is consistent with findings reported by Shafi and Rady, who demonstrated the stability of ocular formulations containing TIM with 2–10% (*w*/*w*) ethyl cellulose, methyl cellulose, and hydroxypropyl cellulose in accelerated conditions (40 °C/75% RH) [32]. In contrast, the spectrum of TIM–HEC mixture stressed with UV/Vis light exhibited notable changes. Specifically, the band at 1708 cm^−1^ attributed to C=O stretching in the maleate salt of TIM disappeared or overlapped with its bands at 1685 and 1670 cm^−1^ due to C–N stretching, consistently with the changes observed for the light-stressed individual TIM. In addition, broadening of the band at 1574 cm^−1^ corresponding to C=C vibrations in TIM maleate was noted (Figure 2A,A1).

**Figure 3:**
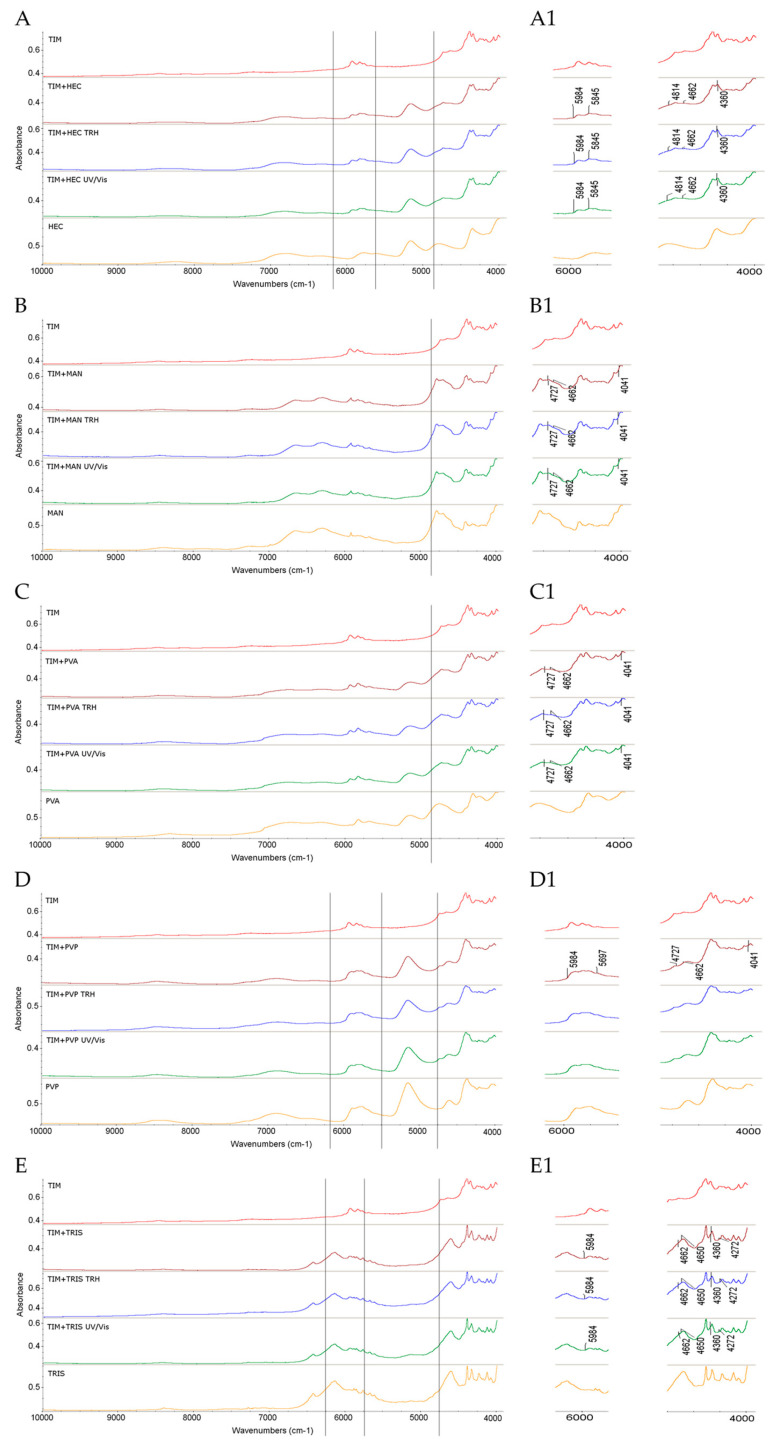
NIR spectra of TIM in binary mixtures with the following: (**A**,**A1**) HEC, (**B**,**B1**) MAN, (**C**,**C1**) PVA, (**D**,**D1**) PVP, and (**E**,**E1**) TRIS in the non-stressed and stressed samples where (**A1**) and onward denote the zoomed parts of the spectra; TRH = the sample stressed with high temperature and high humidity, UV/Vis = the sample stressed with UV/Vis light.

In addition, visible changes were detected in the NIR spectrum of this binary mixture. Specifically, a decrease in the intensity of the bands of TIM at 5984 and 5845 cm^−1^ due to CH_2_ and CH_3_ first overtones, and at 4360 cm^−1^ due to CH_3_ combinations, was observed. What is more, alterations in the band of TIM at 4662 cm^−1^ due to N–H combinations, along with alterations in the shape of the band of HEC at 4814 cm^−1^ due to OH combinations, were clearly seen. These spectral changes suggest the formation of a hydrogen bond between the NH group of the TIM and OH groups of HEC. At the same time, no changes in the NIR spectra were observed after subjecting the samples to high temperature/high humidity or UV/Vis light, indicating that the observed spectral modifications occurred primarily during the initial mixing process (Figure 3A,A1).

When the FT-IR/ATR spectrum of the non-stressed mixture of TIM with MAN was tested, several notable changes were detected. In particular, the band of TIM at 3300 cm^−1^ overlapped with the band of MAN at 3276 cm^−1^, both corresponding to O–H stretching. On the other hand, no additional spectral changes were observed after exposure to high temperature and high humidity. This suggests the potential formation of an intermolecular hydrogen bond involving the OH groups of MAN, even in the absence of applied stress. However, the UV/Vis-stressed mixture exhibited further changes, including broadening of the band of TIM at 1574 cm^−1^ assigned to C=C vibrations and its shift toward a higher wavenumber (1590 cm^−1^), suggesting possible light-induced modifications in the TIM–MAN interactions, particularly in the part of maleate salt. Additionally, the band of TIM at 1708 cm^−1^ corresponding to C=O stretching in maleate disappeared or overlapped with adjacent bands at 1685 and 1670 cm^−1^, consistent with the behavior of UV/Vis-exposed individual TIM (Figure 2B,B1).

The NIR spectrum of the TIM–MAN mixture also showed noticeable changes, including alterations in the shapes of the bands of TIM at 4727, 4662, and 4041 cm^−1^, accompanied by shifts toward higher wavenumbers, suggesting the existence of interactions. Notably, these spectral changes did not intensify following thermal or photolytic stress, indicating that the interactions likely formed during the initial mixing and remained stable under stress conditions (Figure 3B,B1).

Regarding interactions between TIM and MAN, no directly comparable studies were reported in the literature. However, some insights can be drawn from research on bisoprolol fumarate, a β-blocker structurally similar to TIM. In one study, the mixture of bisoprolol fumarate with MAN in a 1:1 ratio (*w*/*w*) was examined in ambient conditions and showed no interactions, based on the corresponding FT-IR spectra [39]. In contrast, when the mixtures of a 1:10 ratio drug-to-excipient ratio (*w*/*w*) were subjected to stress conditions (90 °C for 48 h), quantitative analysis revealed greater degradation of bisoprolol in the presence of MAN compared to other excipients such as sorbitol or PVP, with degradation exceeding 50% [40]. These findings differ somewhat from the results of our thermal degradation studies with TIM; however, differences in drug-to-excipient ratios and the specific chemical nature of these drugs should be considered when interpreting these comparisons.

The FT-IR/ATR spectrum of the non-stressed mixture of TIM with PVA showed no visible changes, compared to the individual components. Similarly, no significant differences were observed after high-temperature/high-humidity stress. However, after UV/Vis light exposure, several spectral changes concerning the bands of TIM were noted. Those included broadening of the band at 1574 cm^−1^, associated with C=C vibrations, distortion of the band at 1708 cm^−1^ corresponding to C=O stretching, and overlapping with adjacent bands at 1685 and 1670 cm^−1^, resulting in a single broad band, consistent with the spectral changes observed for the light-exposed individual TIM (Figure 2C,C1).

The NIR spectra of the TIM–PVA mixture also revealed changes in the shapes of the bands of TIM at 4727, 4662, and 4041 cm^−1^, with their shifts toward higher wavenumbers suggesting changes in the strength of possible hydrogen bonds between TIM and PVA, similarly to the mixtures of TIM with HEC or MAN. These changes appeared independently of stress, and no additional alterations were observed following thermal or photolytic exposure (Figure 3C,C1).

The FT-IR/ATR spectrum of the non-stressed mixture of TIM with PVP showed no visible changes in the absorption patterns, compared to the individual components. Likewise, no significant spectral changes were detected after exposure to high temperature and high humidity. However, following photolytic stress, broadening of the band of TIM at 1574 cm^−1^, attributed to C=C vibrations, was observed. Additionally, the band of TIM at 1708 cm^−1^ corresponding to C=O stretching disappeared or overlapped with its neighboring bands at 1685 and 1670 cm^−1^, resulting in the formation of a single broad absorption band, similarly to the spectrum of individual TIM stressed with light (Figure 2D,D1).

The NIR spectra of this mixture showed changes in the shapes of the bands of TIM at 5984 and 5697 cm^−1^ corresponding to CH_3_ and CH_2_ first overtones, and at 4041 cm^−1^ due to C–H and C–C combinations. Furthermore, the bands of TIM at 4727 cm^−1^ (O–H combinations) and 4662 cm^−1^ (N–H combinations), as well as the band of PVP at 4666 cm^−1^ (N–H combinations), appeared to have shifted toward higher wavenumbers, suggesting new sorts of intramolecular and intermolecular interactions. As was noted above, similar spectral changes were observed in the mixtures of TIM with HEC, MAN, and PVA, probably due to the presence of hydroxyl or polar functional groups in these excipients (Figure 3D,D1).

It is well documented that strong intermolecular interactions, such as hydrogen bonding between drug molecules and polymer chains, play a crucial role in reducing drug mobility and inhibiting crystallization in solid dispersions. Accordingly, PVP can serve as an effective carrier or matrix for various drugs in solid dispersions, helping to maintain the drugs in a stable amorphous state. This property can be particularly beneficial for drugs with low aqueous solubility, as it enhances their dissolution rate and, consequently, their bioavailability [41].

However, some reports on the possible incompatibilities between TIM and PVP are present in the literature. For example, Shafie and Rady observed complete melting of a TIM in a formulation containing 10% (*w*/*w*) PVP after storage at 40 °C/75% RH [32]. Another study investigated the stability of bisoprolol fumarate mixed with PVP in a 1:10 ratio (drug to excipient, *w*/*w*) under stress conditions (90 °C for 48 h), using the HPLC method for quantitative analysis. As a result, notable degradation of the drug in the presence of PVP was observed, although it was less severe than in the presence of MAN, with degradation not exceeding 50% [40]. The above results differ to some extent from those obtained in our thermal degradation studies, where no interactions were observed. However, the differing weight ratios of the components in the tested mixtures, particularly the excess of MAN, must be taken into account.

The most pronounced spectral changes were observed in the binary mixture of TIM and TRIS. In the FT-IR/ATR spectrum of the non-stressed sample, the bands of TIM at 3300 cm^−1^ corresponding to O–H vibrations were greatly reduced or nearly disappeared, likely due to overlap with similar bands of TRIS at 3351 cm^−1^. Additionally, the band at 3088 cm^−1^ attributed to N–H vibrations of TIM also diminished. Further, a partial overlapping and broadening was observed between the C=O stretching band of TIM at 1708 cm^−1^ and the TRIS band at 1626 cm^−1^ due to N–H, probably due to hydrogen bonding. These changes were present in the spectrum of the non-stressed mixture, but upon exposure to high temperature/high humidity or UV/Vis light, these overlapping bands further changed their shapes or nearly disappeared, indicating the formation of new, potentially stronger or more complex interactions (Figure 2E,E1).

Regarding the NIR spectra, the TIM–TRIS mixture exhibited several notable changes compared to the individual components. These included alterations in the shape of the band of TIM at 5984 cm^−1^, overlap of the bands in the 4360–4272 cm^−1^ region due to changes in the CH_3_ and CH_2_ groups, and especially overlapping of bands around 4662 cm^−1^ and 4650 cm^−1^ due to N–H combinations from both compounds. These spectral changes were also observed after high temperature/high humidity and UV/Vis light, although the intensity of the changes did not increase, suggesting that interactions between TIM and TRIS occur during mixing and remain relatively stable under stress conditions (Figure 3E,E1). Interestingly, the behavior of TIM in the presence of TRIS differed from that observed with the other excipients used, especially regarding the C=O group at 1708 cm^−1^, probably due to its stronger interferences with the NH_2_ group of TRIS. The behavior of the bands corresponding to the OH groups of both compounds was also interesting. However, it is important to acknowledge the limitations of the applied analytical methods, as the complexity of the data prevents definitive identification of the exact nature of these interactions.

#### 2.1.3. Results for Binary Mixtures of NAPH

**Figure 4:**
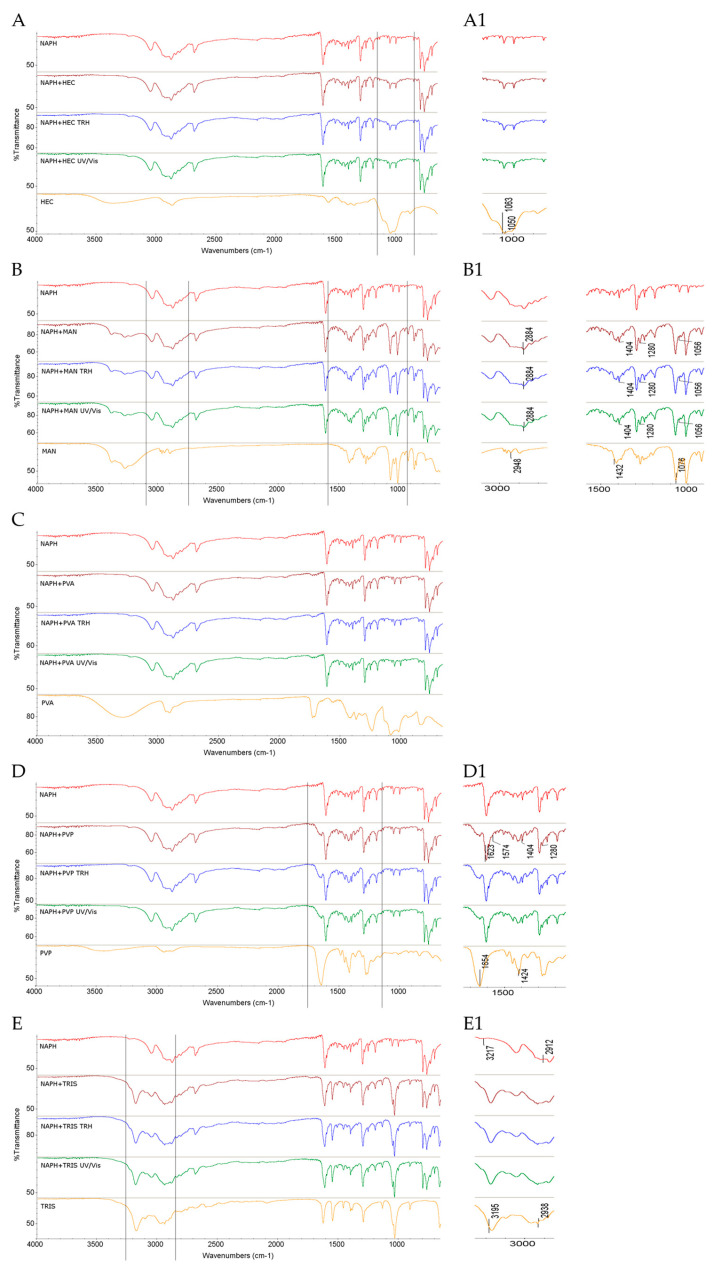
FT-IR/ATR spectra of NAPH in binary mixtures with the following: (**A**,**A1**) HEC, (**B**,**B1**) MAN, (**C**) PVA, (**D**,**D1**) PVP, and (**E**,**E1**) TRIS in the non-stressed and stressed samples, where (**A1**) and onward denote the zoomed parts of the spectra; TRH = the sample stressed with high temperature and high humidity, UV/Vis = the sample stressed with UV/Vis light.

When the FT-IR/ATR spectra of the non-stressed mixture of NAPH with HEC were examined, no visible changes were observed in the absorption pattern of NAPH. Similarly, the samples subjected to high temperature/high humidity and UV/Vis stress did not show additional spectral differences (Figure 4A,A1).

**Figure 5:**
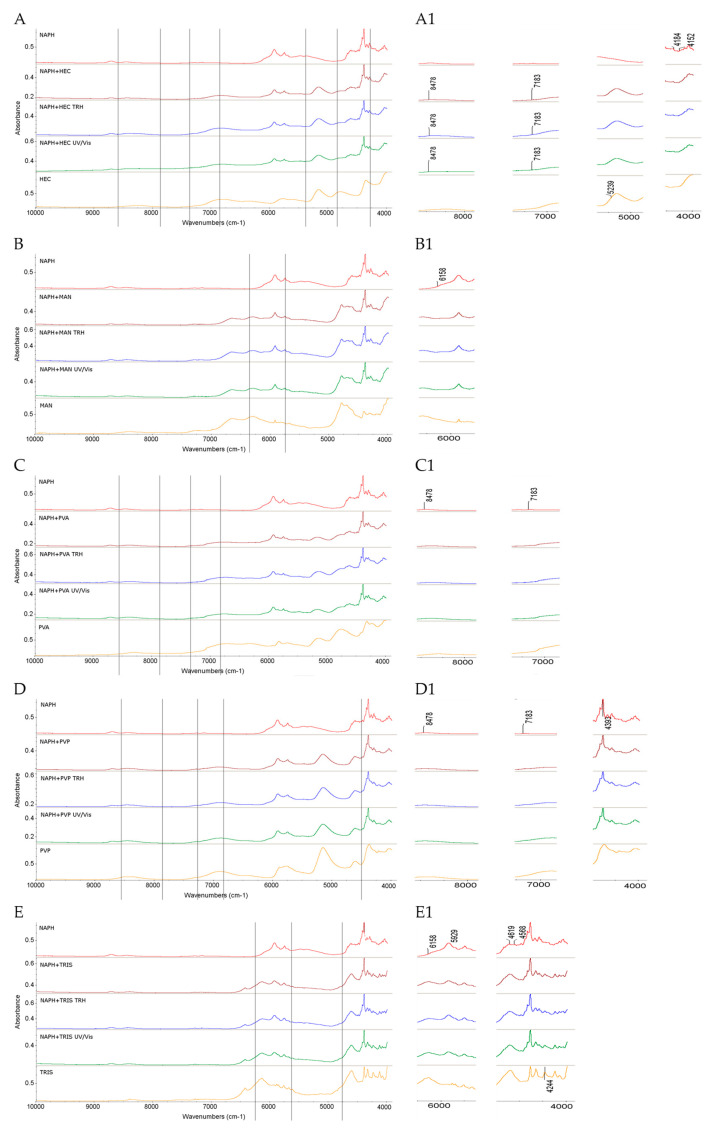
NIR spectra of NAPH in binary mixtures with the following: (**A**,**A1**) HEC, (**B**,**B1**) MAN, (**C**,**C1**) PVA, (**D**,**D1**) PVP, and (**E**,**E1**) TRIS in the non-stressed and stressed samples, where (**A1**) and onward denote the zoomed parts of the spectra; TRH = the sample stressed with high temperature and high humidity, UV/Vis = the sample stressed with UV/Vis light.

Interestingly, the NIR spectra of this binary mixture exhibited more pronounced changes. In the spectrum of the non-stressed mixture, the band of NAPH at 8478 cm^−1^ (CH_2_ and C–H overtones) shifted to lower wavenumbers, accompanied by a change in the shape of the band at 7183 cm^−1^ (C–H first overtone combinations). Simultaneously, the bands of NAPH at 4184 and 4152 cm^−1^ (CH_2_ and C–H overtones) were nearly absent. Additionally, the band at 5239 cm^−1^ due to O–H combinations from HEC clearly disappeared. Similar spectral changes were observed in the mixtures of NAPH with HEC subjected to high temperature/high humidity and UV/Vis light stress (Figure 5A,A1). Thus, interactions such as intermolecular hydrogen bond formation could be suggested between the N–H groups of NAPH and the O–H groups of HEC, although the obtained FT-IR/ATR spectra did not confirm this interaction clearly.

Regarding the FT-IR/ATR spectra of binary mixtures of NAPH with MAN, similar changes were observed in the non-stressed and stressed samples. Clear overlapping of NAPH bands at 2884 cm^−1^ (C–H stretching in the methylene group), 1404 cm^−1^ (C–C stretching in the five-membered ring), 1280 cm^−1^ (C–N stretching), and 1056 cm^−1^ (C–H bending) with corresponding MAN bands was evident. Additionally, MAN bands at 2948 cm^−1^ and 1432 cm^−1^ (both due to C–H bending vibrations), as well as the band at 1076 cm^−1^ (C–O stretching), overlapped with the corresponding NAPH bands. All these changes were consistently present in the spectra of the stressed samples (Figure 4B,B1). Bearing in mind possible interactions, of particular interest were the changes involving the C–O vibrations from MAN at 1076 cm^−1^, although the nature of these possible interactions remains difficult to characterize. Analysis of the NIR spectra revealed some changes, including the disappearance of bands of NAPH at 6158 cm^−1^ (C–H aromatic ring overtones), observed both before and after exposure to stress conditions (Figure 5B,B1).

The FT-IR/ATR spectra of the NAPH and PVA mixture did not show notable changes in the non-stressed and stressed mixtures (Figure 4C). However, the NIR spectra revealed some alterations, including the disappearance of NAPH bands at 8478 cm^−1^ and 7183 cm^−1^ due to CH_2_ and C–H second overtones, and C–H first overtone combinations in the non-stressed and stressed samples (Figure 5C,C1).

In contrast, the FT-IR/ATR spectrum of the NAPH and PVP mixture displayed several changes even without stressing. Notably, the NAPH band at 1623 cm^−1^ due to N–H stretching overlapped with the PVP band at 1654 cm^−1^ due to C=O stretching, while the band at 1404 cm^−1^ of NAPH overlapped with the PVP band at 1424 cm^−1^. Additionally, changes were observed in bands near 1280 cm^−1^, corresponding to C–N stretching in the imidazoline ring of NAPH (Figure 4D,D1). These spectral features strongly suggest the formation of new intermolecular hydrogen bonds, consistent with known interactions between the NH and C=O groups of adjacent molecules [41]. Supporting this, a theoretical study from the literature describes intermolecular complexes formed between PVP and various imidazolidine derivatives [42].

In the present study, interactions between NAPH and PVP were further confirmed by the disappearance of NAPH bands at 8478 cm^−1^ (CH_2_ and C–H second overtones) and 7183 cm^−1^ (C–H first overtones) in the NIR spectrum. Additionally, the band at 4393 cm^−1^ showed a noticeable decrease in its intensity. These changes were evident both without any stress and after exposure to high temperature/humidity and UV/Vis light (Figure 5D,D1).

Regarding the mixture of NAPH with TRIS, its FT-IR/ATR spectrum showed some changes that appeared without any stressing and persisted after both stress conditions. These included overlapping some bands of NAPH, at 3217 cm^−1^ due to N–H stretching and 2912 cm^−1^ due to C–H stretching with nearby bands of TRIS (Figure 4E,E1). Analysis of the NIR spectra revealed even more pronounced changes, which were present before and after exposure to temperature/humidity and light stress. Specifically, the disappearance of the bands of NAPH at 6158 and 5929 cm^−1^ (C–H aromatic ring overtones) and at 4619 and 4568 cm^−1^ (N–H combinations) was noted. Clear changes were also observed in the TRIS spectrum at 4244 cm^−1^, where the shape of bands was noticeably altered (Figure 5E,E1). Similar changes in the NIR spectrum, engaging the bands of both compounds due to N–H combinations, were observed for the mixture of TIM and TRIS.

Taking the above results into account, visual analysis of the spectra of NAPH in its mixtures with HEC and PVP, and to a lesser extent with MAN and TRIS, proves challenging. Moreover, similar studies are lacking in the literature, although intermolecular hydrogen bonding occurring between imidazolidine derivatives and PVP was reported [42]. Nevertheless, the current findings suggest that NAPH is relatively resistant to the presence of excipients. This may be attributed to the absence of highly reactive groups within the NAPH molecule (Figure S1, Supplementary Materials).

#### 2.1.4. Results for Binary Mixtures of DIF

**Figure 6:**
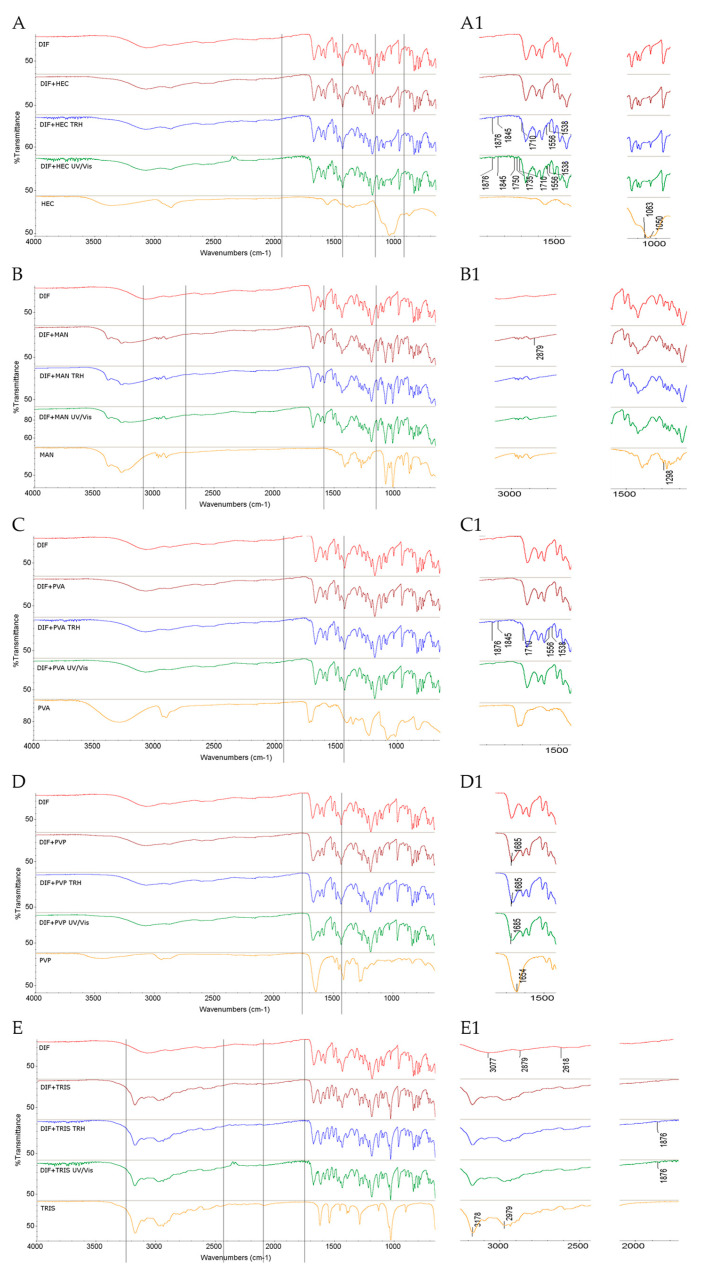
FT-IR/ATR spectra of DIF in binary mixtures with the following: (**A**,**A1**) HEC, (**B**,**B1**) MAN, (**C**,**C1**) PVA, (**D**,**D1**) PVP, and (**E**,**E1**) TRIS in the non-stressed and stressed samples, where (**A1**) and onward denote the zoomed parts of the spectra; TRH = the sample stressed with high temperature and high humidity, UV/Vis = the sample stressed with UV/Vis light.

Examination of the FT-IR/ATR spectrum of the non-stressed mixture of DIF and HEC showed no visible changes in the absorption pattern of DIF. However, after exposure to high temperature and high humidity, noticeable changes were observed. It was clearly seen that the DIF band at 1876 cm^−1^, attributed to C–H deformation, changed its shape. Additionally, several small bands of DIF appeared in the 1845–1710 cm^−1^ region. Alterations were also noted in the spectrum of the UV/Vis-stressed sample, including distinct new bands at 1750, 1735, 1556, and 1538 cm^−1^ (Figure 6A,A1).

**Figure 7:**
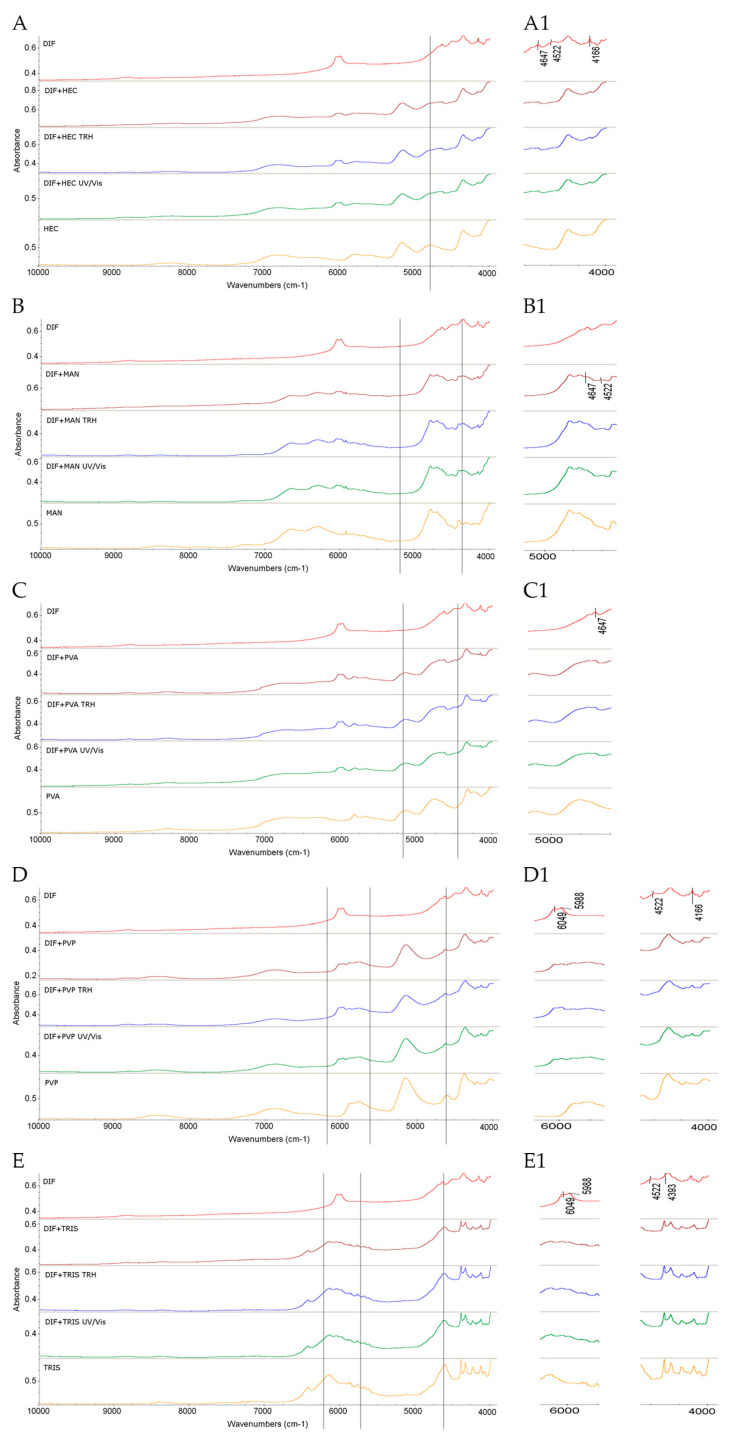
NIR spectra of DIF in binary mixtures with the following: (**A**,**A1**) HEC, (**B**,**B1**) MAN, (**C**,**C1**) PVA, (**D**,**D1**) PVP, and (**E**,**E1**) TRIS in the non-stressed and stressed samples, where (**A1**) and onward denote the zoomed parts of the spectra; TRH = the sample stressed with high temperature and high humidity, UV/Vis = the sample stressed with UV/Vis light.

In the NIR spectrum of the non-stressed mixture, the bands of DIF at 4647, 4522, and 4166 cm^−1^ due to C–H and C=O, as well as CH_2_ and C–H second overtone combinations, were absent. Similar changes were detected in spectra of the mixtures subjected to high temperature/high humidity and UV/Vis light (Figure 7A,A1). Notably, the most interesting changes involved the C=O group of DIF.

In the spectrum of the binary mixture of DIF with MAN, the most notable change included a shape alteration of the DIF band at 2879 cm^−1^ attributed to O–H stretching in the carboxylic group. A similar change was observed due to the overlap of the DIF band at 1298 cm^−1^ related to O–H deformation with the corresponding MAN band at 1298 cm^−1^. These changes were noted under ambient conditions and after exposing the samples to temperature/humidity or UV/Vis light stress (Figure 6B,B1). Notable changes were also observed in the NIR spectra of this mixture. In the non-stressed and stressed samples, the DIF bands at 4647 cm^−1^ and 4522 cm^−1^ due to C–H and C=O combinations were affected (Figure 7B,B1). Considering these alterations, interactions involving the C=O group of DIF could be suggested, similarly to those observed in the mixture with HEC.

The FT-IR/ATR spectrum of the mixture of DIF and PVA showed no visible changes after mixing and storage under ambient conditions, or after UV/Vis light exposure. However, after high temperature/high humidity stress, some changes became evident. Specifically, the DIF band at 1876 cm^−1^ attributed to C–H deformation exhibited a shape change, and several small bands appeared in the ranges of 1845–1710 cm^−1^ and of 1556–1538 cm^−1^, similarly to the DIF–HEC mixture (Figure 6C,C1). On the other hand, the NIR spectra of DIF–PVA mixture did not show notable changes under either ambient or stressed conditions, except for the lack of the DIF band at 4647 cm^−1^ (C–H and C=O combinations), which was observed consistently across all conditions (Figure 7C,C1).

In the FT-IR/ATR spectrum of the mixture of DIF and PVP, distinct changes were observed immediately after mixing. The most prominent was the alteration in the shape of the DIF band at 1685 cm^−1^ attributed to C=O stretching of the carboxylic group, likely due to the overlap with the PVP band at 1654 cm^−1^, which also corresponds to C=O stretching. These changes were consistently present in all samples, both the non-stressed and stressed ones (Figure 6D,D1). According to the literature, solid dispersions and physical mixtures of DIF with PVP in a 7:3 drug-to-polymer ratio form intermolecular hydrogen bonds, as evidenced by broadening of the band corresponding to the DIF C=O group in the respective FT-IR spectrum, although such interactions were not observed in a physical mixture of similar composition [37]. Similar behavior was also reported for ibuprofen, another acidic NSAID, which interacted with PVP in solid dispersions similarly to DIF, primarily via hydrogen bonding between its carboxylic group and the nitrogen atom in the pyrrolidone ring of PVP [43].

In the NIR spectra analyzed in the present study, the DIF bands at 4522 cm^−1^ and 4166 cm^−1^ due to C–H and C=O combinations were absent in the spectrum of the mixture under ambient conditions. Further changes were observed after high temperature/high humidity and UV/Vis light stress, including the disappearance or shape alteration of DIF bands at 6049 cm^−1^ and 5988 cm^−1^, which correspond to the first overtones of C–H vibrations in the aromatic ring (Figure 7D,D1). Based on visual analysis of our spectral data and the available literature, interactions involving the C=O group of DIF could be suggested; however, clear evidence for the formation of intermolecular hydrogen bonds could not be confirmed.

In the FT-IR/ATR spectrum of the DIF and TRIS binary mixture, visible changes were observed after mixing and storing under ambient conditions. Notable overlapping occurred between the DIF band at 3077 cm^−1^ (C–H stretching in the aromatic ring) and the TRIS band at 2979 cm^−1^ (C–H stretching). Additionally, the shapes of the DIF bands at 2879 cm^−1^ and 2618 cm^−1^, both corresponding to O–H stretching of the carboxylic group, were altered. The DIF band at 3077 cm^−1^ also shifted to higher wavenumbers and overlapped with the TRIS-associated band at 3178 cm^−1^ due to N–H stretching. Upon exposure to stress conditions, further alterations were observed, including a notable change in the shape of the DIF band at 1876 cm^−1^ (Figure 6E,E1). Moreover, the NIR spectrum of the non-stressed mixture showed the disappearance of or alteration in several DIF bands, including those at 6049 and 5988 cm^−1^ (C–H first overtones in the aromatic ring) and at 4647, 4522, and 4393 cm^−1^ (C–H and C=O combinations). These changes were also evident under stress conditions, although their intensity did not increase significantly (Figure 7E,E1).

Considering the observed spectral changes and the distinct chemical nature of acidic DIF and basic TRIS, potential chemical interactions between the two can be proposed, especially under high-temperature and high-humidity conditions. In a study from the literature, physical mixtures and coprecipitates of acidic NSAID ketoprofen with basic excipients, including TRIS, were investigated using differential scanning calorimetry (DSC) and FT-IR spectroscopy, suggesting hydrogen bond formation and electrostatic interactions of ketoprofen with TRIS. Additionally, TRIS was highlighted for its role as a potential solubilizer, and its capacity to form salts was highlighted [29]. Another study explored interactions between acidic indometacin and TRIS during hot-melt extrusion of a 1:1 molar ratio. As a result, spectroscopic analyses (IR and NMR) demonstrated the possibility of in situ salt formation [44].

Taking all these findings into account, it can be concluded that visual evaluation of potential interactions of the studied APIs (TIM, NAPH, and DIF) and excipients (HEC, MAN, PVA, PVP, and TRIS) is subjective to some extent. This limitation could be overcome by applying chemometric analysis to spectral data, enabling more objective and reliable interpretations of the interactions.

### 2.2. Chemometric Analysis of FT-IR/ATR and NIR Data

To extract the maximum relevant information from the FT-IR/ATR and NIR spectra of the studied APIs (TIM, NAPH, and DIF), excipients (HEC, MAN, PVA, PVP, and TRIS), and their binary physical mixtures subjected to high temperature/high humidity and UV/Vis light, two chemometric methods were employed—PCA and HCA. These unsupervised, multivariate statistical methods enable advanced data interpretation. PCA, in particular, reduces the dimensionality of large datasets and presents the results in a simplified, typically two-dimensional, score scatter plot for easier visualization. This makes it possible to interpret the obtained measurement data with minimal loss of information. In turn, HCA operates by identifying similarities and differences among the studied samples. The results are presented as a dendrogram, which visually illustrates the tendency of the samples to form clusters or groups characterized by high internal similarity based on specific spectral features, while remaining as distinct as possible from other clusters [45].

The data in Table 1 show that the percentage of variance explained by the first principal component (PC1) was always greater for NIR spectra than for FT-IR/ATR spectra. Moreover, with the exception of the matrix involving mixtures of NAPH with excipients, the variance explained by PC1 was found to be high for the other mixtures, ranging from more than 92% to more than 95%. This suggests that for the three matrices involving mixtures of TIM, NAPH, and DIF with five excipients, the location of the samples under study can be considered in a one-dimensional system, i.e., along the PC1 axis. Since the total variance in PCA is 100%, and each subsequent PC explains a smaller percentage of the variance than the preceding PC, the contribution of PC2 and PC3 to the distribution of samples on the PCA score scatter plot is negligible.

The results of applying two chemometric methods, PCA and HCA, to evaluate the effects of high temperature/high humidity and UV/Vis light on the stability of TIM, NAPH, and DIF are summarized in Table 2. Analysis of these data revealed that the two spectroscopic techniques, FT-IR/ATR and NIR, differ significantly in their ability to detect the extent of undesirable changes in the examined APIs. The terms strong (+++), medium (++), weak (+) or no impact (−) were used to assess the degree to which the samples were affected by the stress conditions. These terms cannot be substituted by numerical values, since both chemometric methods lead to generalization of the processed measurement data, only indicating certain clustering tendencies of the samples. Similar terminology is commonly accepted in FT-IR/ATR, NIR, Raman, and other spectroscopic techniques. The different intensity and shape of bands in IR spectra are referred to as strong, medium, weak, broad, or sharp.

Generalizing from the chemometric results, it can be concluded that NIR spectra indicate a greater effect of stress conditions on the stability of TIM, NAPH, and DIF than FT-IR/ATR spectra, with UV/Vis light having a greater effect on APIs than high-temperature/high-humidity stress. In addition, it is important to note that these two chemometric methods differ in their approach to processed spectroscopic data. PCA reduces multidimensionality while HCA searches for similarities, which often creates significant differences in the interpretation of the same FT-IR/ATR or NIR spectra [45].

**Figure 8:**
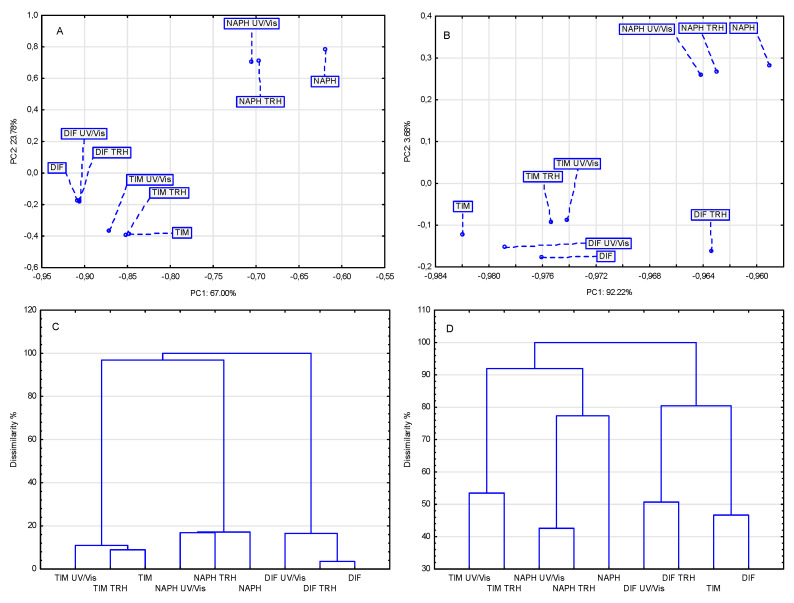
PCA score scatter plots for FT-IR/ATR (**A**) and NIR (**B**) spectral data as well as HCA dendrograms for FT-IR (**C**) and NIR (**D**) spectral data of the non-stressed and stressed samples of TIM, NAPH, and DIF.

Figure 8 presents the PCA score scatter plot and HCA dendrogram developed from the FT-IR/ATR and NIR spectral data of individual APIs. The PCA plot (Figure 8A) shows three clusters, each grouping the API and its samples exposed to stress conditions. With the exception of the cluster with NAPH, the other clusters occupy similar values of PC1 and PC2, indicating no or weak effect of stress conditions on individual API stability. The HCA dendrogram shown in Figure 8C leads to a similar conclusion. The dendrogram indicates a lack of similarity between TIM, NAPH, and DIF, as evidenced by the linkage of the three clusters containing the examined APIs only at the 98% and 100% dissimilarity levels. In contrast, the individual APIs and their samples exposed to high temperature/high humidity and UV/Vis light form clusters at levels ranging from 4% to 18% dissimilarity. This confirms that the FT-IR/ATR spectra of APIs stored under ambient conditions and those exposed to stress conditions are not significantly different from each other, suggesting that there is no or weak effect of stress conditions on API stability.

The PCA score scatter plot for data from NIR spectra of TIM, NAPH, and DIF under and without stress conditions shows that some of the samples are located at a considerable distance from the others, indicating a medium or strong effect of stress conditions on their stability (Figure 8B). A slightly different finding is reached by the HCA dendrogram, which indicates that, for example, the NIR spectra of TIM samples exposed to high temperature/high humidity and UV/Vis light are 100% dissimilar to the NIR spectrum of TIM not exposed to stress conditions (Figure 8D). Regarding the samples of DIF and NAPH exposed to stress conditions, they show dissimilarity levels of 81% and 74%, respectively, compared to the spectra of unexposed drugs. Such grouping of these samples exposed to stress conditions would indicate their thermal degradation.

The results of applying PCA and HCA to interpret the FT-IR/ATR and NIR spectra of individual excipients (HEC, MAN, PVA, PVP, and TRIS) after exposure to high temperature/high humidity and UV/Vis light are summarized in Table S2 (Supplementary Materials). Comparing the results obtained with those compiled in Table 2, it can be concluded that the examined excipients are more resistant to stress conditions than APIs. As with the APIs, the results varied depending on the spectroscopic technique employed. In general, NIR spectra indicated a greater effect of stress conditions on the stability of excipients than the effect observed with FT-IR/ATR spectra. It was also shown that excipients were slightly more affected by UV/Vis light than by high temperature/high humidity. Additionally, the PCA and HCA results for the excipients showed greater consistency with each other than the corresponding results obtained for the APIs.

**Figure 9:**
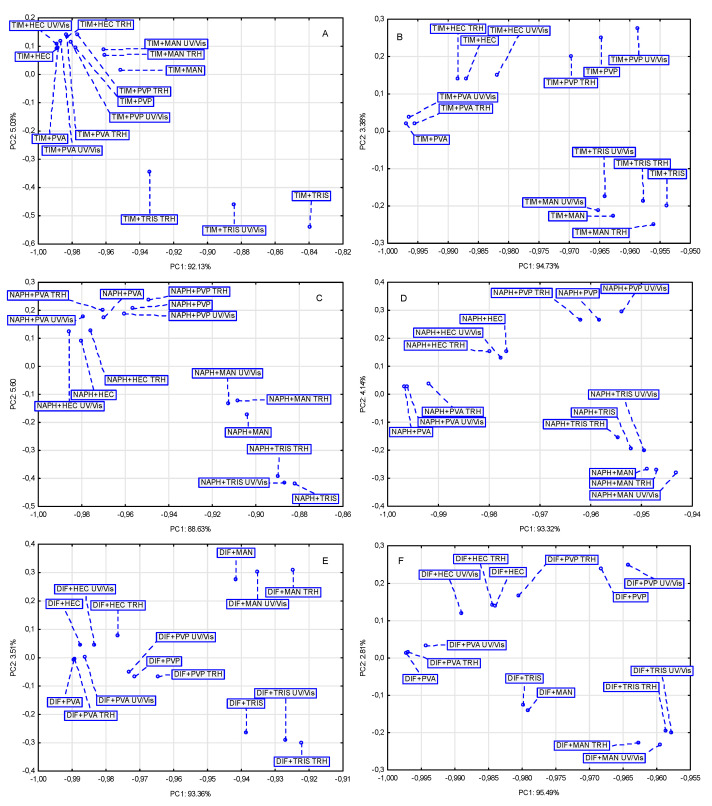
PCA score scatter plots for FT-IR/ATR (**A**,**C**,**E**) and NIR (**B**,**D**,**F**) spectral data of the non-stressed and stressed binary physical mixtures of TIM (**A**,**B**), NAPH (**C**,**D**), and DIF (**E**,**F**) with excipients, HEC, MAN, PVA, PVP, and TRIS.

**Figure 10:**
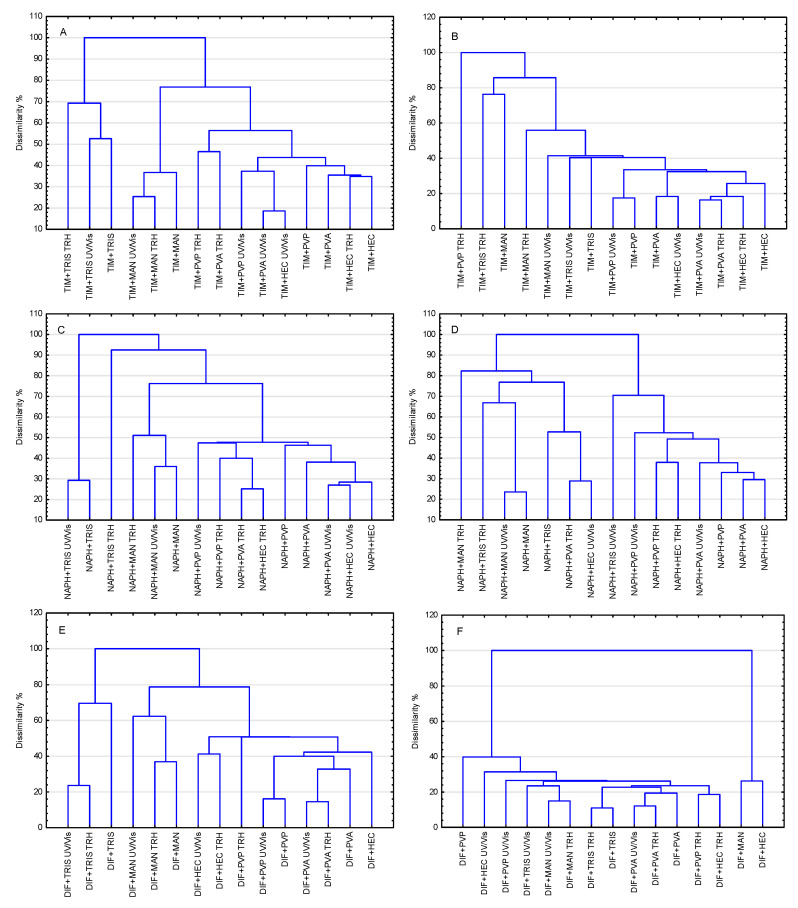
HCA dendrograms for FT-IR/ATR (**A**,**C**,**E**) and NIR (**B**,**D**,**F**) spectral data of the non-stressed and stressed binary physical mixtures of TIM (**A**,**B**), NAPH (**C**,**D**), and DIF (**E**,**F**) with excipients, HEC, MAN, PVA, PVP, and TRIS.

The results obtained for the binary mixtures are illustrated graphically in Figure 9 and Figure 10, with detailed data compiled in Table 3, Table 4 and Table 5. Analysis of these data indicated that undesirable changes occurred to varying degrees in all examined mixtures under the applied stress conditions. It was found that the mixtures of NAPH with excipients proved to be the least sensitive to the influence of stress conditions. The relationships identified with PCA and HCA between NAPH mixtures stored under ambient conditions and influenced by stress conditions are shown in Figure 9C and Figure 10C (for FT-IR spectra) and Figure 9D and Figure 10D (for NIR spectra). The situation was much worse for mixtures of TIM (Figure 9A and Figure 10A for FT-IR spectra and Figure 9B and Figure 10B for NIR spectra) and DIF (Figure 9E and Figure 10E for FT-IR spectra and Figure 9F and Figure 10F for NIR spectra) with all excipients, which exhibited comparable but much weaker resistance to stress factors.

It should be noted that the sensitivity of the binary mixtures to stress conditions was strongly influenced by the excipient. However, some regularities were detected that extend to all the mixtures studied. Among the five excipients, HEC, MAN, PVA, PVP, and TRIS, the mixtures containing TRIS exhibited the lowest resistance to stress conditions, regardless of whether TIM, NAPH, or DIF was the active ingredient. MAN was another excipient whose presence in the mixtures contributed to reduced resistance of the APIs to stress conditions. These results indicate that the studied APIs are most compatible with HEC, PVA, or PVP for potential drug formulations. In the presence of these three excipients, mixtures with TIM, NAPH, and DIF were relatively resistant to high temperature/high humidity and UV/Vis light stress.

In-depth analysis of the obtained spectral data led to the general conclusion that the use of chemometric methods, i.e., PCA and HCA, significantly enhances the interpretation of the obtained spectra. The use of chemometric methods makes it possible to include subtle changes in the FT-IR/ATR and NIR spectra, which could not be detected using visual interpretation, in the evaluation of the effect of mixing the components or stress conditions on the studied samples. These are reflected by absorption values recorded every 0.5 cm^–1^ in the case of FT-IR/ATR spectra and every 4 cm^–1^ in the case of NIR spectra.

The results presented in Section 2.1. indicated the absence of any visible changes in the NIR spectra of the non-stressed and stressed samples of TIM, NAPH, and DIF. Significant changes were observed in the FT-IR/ATR spectra only for TIM samples exposed to UV/Vis light, while no visible changes were detected in the FT-IR/ATR spectra of the other APIs. On the other hand, the results of PCA and HCA, compiled in Table 1 and illustrated graphically in Figure 8, provide a new perspective on the interpretation of the NIR and FT-IR/ATR spectra of the studied APIs. The absence of any visible changes in the NIR spectra of the non-stressed and stressed APIs may be due to the fact that the NIR spectra primarily reflect oscillatory vibrations associated with the presence of functional groups such as –CH, –NH, –OH, and –SH, and the bands illustrating these vibrations usually overlap to form spectra with broad bands. As a result, NIR spectra are relatively band-poor, making their interpretation more challenging compared to FT-IR/ATR spectra. However, NIR radiation possesses higher energy than mid-infrared radiation, allowing it to penetrate deeper into sample layers. This enables the acquisition of more comprehensive information about the molecular and physical structure of the material, while also minimizing issues such as sample inhomogeneity. At the same time, both of the above-mentioned properties of NIR spectroscopy highlight the importance of incorporating chemometrics into the interpretation of NIR, as well as FT-IR/ATR spectra, to extract the most valuable information [46,47].

By combining the visual interpretation of NIR and FT-IR/ATR spectra with chemometric analyses, specifically PCA scatter plots and HCA dendrograms, reliable information was obtained on the effects of stress conditions (high temperature/high humidity and UV/Vis light) on the studied APIs and their mixtures with five various excipients. Notably, some interactions between the drugs and excipients were also detected under ambient conditions, particularly in mixtures containing TRIS, MAN, and PVP, identifying these as the most reactive excipients. However, it must be noted that some of these interactions could be beneficial from a pharmaceutical perspective, enhancing the solubility and bioavailability of respective APIs.

## 3. Materials and Methods

### 3.1. Chemicals

Pharmaceutical-grade standards of timolol (TIM, (2S)-1-(tert-butylamino)-3-[(4-morpholin-4-yl-1,2,5-thiadiazol-3-yl)oxy]propan-2-ol, (Z)-but-2-enedioic acid), naphazoline (NAPH, 2-(naphthalen-1-ylmethyl)-4,5-dihydro-1H-imidazole, hydrochloride), diflunisal (DIF, 5-(2,4-difluorophenyl)-2-hydroxybenzoic acid), hydroxyethyl cellulose (HEC), mannitol (MAN), poly(vinyl alcohol) (PVA), poly(vinylpyrrolidone) (PVP), and Tris HCl (TRIS) were purchased from Sigma-Aldrich (St. Louis, MO, USA). Physical binary mixtures of these drugs and excipients were prepared by mixing the components at a 1:1 (*w*/*w*) ratio using an agate mortar [48,49]. Then each individual drug, individual excipient, and their binary mixtures were weighed into 20 mg portions and evenly dispersed in standardized small flat vessels, forming the sample layers, which are approximately 3 mm thick.

### 3.2. Instrumentation

A KBF-LQC climate chamber (Binder GmbH, Tuttlingen, Germany) was used to apply high-temperature and high-humidity stress conditions. Photolytic stress was applied using a Suntest CPS PLUS solar simulation chamber (Atlas, Linsengericht, Germany), which provides the controlled UV/Vis light exposure in the wavelength range of 300–800 nm. FT-IR/ATR and NIR spectra were recorded using a Nicolet 6700 spectrometer (Thermo Scientific, Waltham, MA, USA), equipped with a Smart iTR™ ATR sampling accessory for FT-IR/ATR and a Near-IR integrating sphere for NIR measurements. Spectral data were processed and analyzed using OMNIC v. 8.1 software (Thermo Scientific).

### 3.3. Forced Degradation

The first set of prepared samples was placed in the climate chamber and exposed to stress conditions of 70 °C and 80% RH for 35 h. The second set was placed in the light chamber and irradiated with a total energy dose of 94.510 kJ/m^2^. The stress conditions used corresponded to those recommended for forced degradation by official guidelines, which require the use of temperature and humidity above accelerated conditions and extreme photolytic tests [7,50]. The remaining samples, which served as the non-stressed controls, were stored in a desiccator under ambient conditions (23 ± 2 °C) and protection from UV/Vis light. After completion of the stress treatments, all samples were transferred to a desiccator and stored under controlled conditions until analysis.

### 3.4. FT-IR/ATR and NIR Spectrometry

Following the background spectrum acquisition, the samples were analyzed in the range of 4000–800 cm^−1^ for FT-IR/ATR and 10,000–4000 cm^−1^ for NIR spectroscopy. Each sample was measured four times, and the spectrum used for further analysis represented the average of these four scans. The spectra of the non-stressed and stressed individual drugs, TIM, NAPH, DIF, and five excipients, HEC, MAN, PVA, PVP, and TRIS, were compared with the corresponding non-stressed controls. In the next phase of the study, binary mixtures of each drug with each excipient, both the non-stressed and stressed, were analyzed using FT-IR/ATR and NIR spectroscopy and compared.

### 3.5. Chemometric Calculations

Two chemometric methods, Principal Component Analysis (PCA) and Hierarchical Cluster Analysis (HCA), were employed to support the interpretation of FT-IR/ATR and NIR spectral data. Data matrices were constructed using spectral information from FT-IR/ATR and NIR spectra of drugs, excipients, and their corresponding physical mixtures. For FT-IR/ATR analysis, the matrices were composed with absorption values recorded at 0.5 cm^−1^ intervals across the 4000–650 cm^−1^ range. For NIR spectra, absorption values recorded at 4 cm^−1^ intervals within the 10,000–4000 cm^−1^ range were taken into account.

All chemometric calculations were performed using Statistica 13.3 (TIBCO Software Inc., Palo Alto, CA, USA) based on a correlation matrix. For PCA, the score scatter plots were generated in a two-dimensional system using the first and second principal components (PC1 and PC2), without rotation. In HCA, Ward’s method was applied in combination with Euclidean distance to assess similarities between samples. The results were visualized as a dendrogram, where the vertical axis represents the percentage of dissimilarity between the analyzed spectra.

## 4. Conclusions

In the present study, interactions between three drugs with differing physicochemical properties, TIM, NAPH, and DIF, and five excipients potentially suitable for their topical and oral formulations, were investigated under forced degradation conditions. Two spectroscopic techniques, FT-IR/ATR and NIR, were employed, both of which are non-destructive, cost-effective, and rapid, and require minimal sample preparation. To enhance data interpretation, chemometric analyses, including PCA and HCA, were applied. This combined approach significantly reduced the subjectivity associated with visual interpretation of the spectral data, enabling effective detection of potential interactions between the studied APIs and excipients, as well as their susceptibility to degradation. Notably, clear interactions between TIM, NAPH, and DIF with MAN and TRIS were identified, whereas mixtures containing NAPH and excipients showed the highest resistance to forced degradation. From a practical standpoint, these findings can inform the development of potential new formulations containing the studied drugs. Early identification of stability issues may help prevent costly and time-consuming failures in later stages of pharmaceutical development.

## Figures and Tables

**Table 1:** Percent of variability explained by the first principal components (PC1, PC2, and PC3).

Matrices	IR Spectra	PC1 (%)	PC2 (%)	PC3 (%)
Active pharmaceutical ingredients	FT-IR/ATR	67.00	23.78	8.97
	NIR	92.22	3.68	1.94
Excipients	FT-IR/ATR	60.59	17.44	13.71
	NIR	88.15	8.16	2.97
TIM mixtures with excipients	FT-IR/ATR	92.13	5.03	1.85
	NIR	94.73	3.38	1.33
NAPH mixtures with excipients	FT-IR/ATR	88.63	5.60	4.04
	NIR	93.32	4.14	1.88
DIF mixtures with excipients	FT-IR/ATR	93.36	3.51	3.07
	NIR	95.49	2.81	1.21

**Table 2:** Effect of high temperature/high humidity (70 °C/80% RH) and UV/Vis light (94.510 kJ/m^2^) on active pharmaceutical ingredients, TIM, NAPH, and DIF.

Active Pharmaceutical Ingredients	IR Spectra	High Temperature/ High Humidity		UV/Vis Light	
		PCA	HCA	PCA	HCA
TIM	FT-IR/ATR	–	–	+	+
	NIR	++	+++	++	+++
NAPH	FT-IR/ATR	++	+	++	+
	NIR	+	+++	++	+++
DIF	FT-IR/ATR	–	–	–	+
	NIR	+++	+++	+	+++

The influence of stress conditions: +++ strong, ++ medium, + weak, – no impact.

**Table 3:** Effect of high temperature/high humidity (70 °C/80% RH) and UV/Vis light (94.510 kJ/m^2^) on binary mixtures of TIM with excipients, HEC, MAN, PVA, PVP, and TRIS.

Binary Mixtures of TIM with Excipients	IR Spectra	High Temperature/ High Humidity		UV/Vis Light	
		PCA	HCA	PCA	HCA
TIM and HEC	FT-IR/ATR	+	++	–	++
	NIR	+	++	++	++
TIM and MAN	FT-IR/ATR	++	++	++	++
	NIR	++	+++	+	+++
TIM and PVA	FT-IR/ATR	+	+++	+	++
	NIR	+	++	+	++
TIM and PVP	FT-IR/ATR	+	+++	+	++
	NIR	++	+++	++	+
TIM and TRIS	FT-IR/ATR	+++	+++	++	+++
	NIR	++	+++	+++	+++

The influence of stress conditions: +++ strong, ++ medium, + weak, – no impact.

**Table 4:** Effect of high temperature/high humidity (70 °C/80% RH) and UV/Vis light (94.510 kJ/m^2^) on binary mixtures of NAPH with excipients, HEC, MAN, PVA, PVP, and TRIS.

Binary Mixtures of NAPH with Excipients	IR Spectra	High Temperature/ High Humidity		UV/Vis Light	
		PCA	HCA	PCA	HCA
NAPH and HEC	FT-IR/ATR	+	++	+	++
	NIR	+	+++	+	+++
NAPH and MAN	FT-IR/ATR	+	+++	+	++
	NIR	+	+++	++	++
NAPH and PVA	FT-IR/ATR	+	++	+	++
	NIR	+	+++	–	++
NAPH and PVP	FT-IR/ATR	+	++	+	++
	NIR	+	++	+	+++
NAPH and TRIS	FT-IR/ATR	+	+++	+	+++
	NIR	+	+++	+	+++

The influence of stress conditions: +++ strong, ++ medium, + weak, – no impact.

**Table 5:** Effect of high temperature/high humidity (70 °C/80% RH) and UV/Vis light (94.510 kJ/m^2^) on binary mixtures of DIF with excipients, HEC, MAN, PVA, PVP, and TRIS.

Binary Mixtures of DIF with Excipients	IR Spectra	High Temperature/ High Humidity		UV/Vis Light	
		PCA	HCA	PCA	HCA
DIF and HEC	FT-IR/ATR	++	+++	+	+++
	NIR	–	+++	++	+++
DIF and MAN	FT-IR/ATR	++	++	+	+++
	NIR	+++	+++	+++	+++
DIF and PVA	FT-IR/ATR	–	++	+	++
	NIR	–	+	+	+
DIF and PVP	FT-IR/ATR	+	+++	+	+
	NIR	+++	++	++	++
DIF and TRIS	FT-IR/ATR	++	+++	++	+++
	NIR	+++	+	+++	++

The influence of stress conditions: +++ strong, ++ medium, + weak, – no impact.

## Data Availability

The original contributions presented in this study are included in the article/Supplementary Material. Further inquiries can be directed to the corresponding author.

## Supplementary Materials

The following supporting information can be downloaded at https://www.mdpi.com/article/10.3390/molecules30183807/s1, Figure S1: The chemical structures of the drugs and excipients being tested; Figure S2: The spectra of the non-stressed and stressed samples of the drugs: (A) FT-IR/ATR spectra of NAPH and DIF; (B) NIR spectra of TIM, NAPH, and DIF; TRH = the sample stressed with high temperature and high humidity, UV/Vis = the sample stressed with UV/Vis light; Figure S3: FT-IR/ATR spectra of (A) hydroxyethyl cellulose (HEC), (B) mannitol (MAN), (C) poly(vinyl alcohol) (PVA), (D) poly(vinylpyrrolidone) (PVP), and (E) Tris HCl (TRIS) in the non-stressed and stressed samples; TRH = the sample stressed with high temperature and high humidity, UV/Vis = the sample stressed with UV/Vis light; Figure S4: NIR spectra of (A) hydroxyethyl cellulose (HEC), (B) mannitol (MAN), (C) poly(vinyl alcohol) (PVA), (D) poly(vinylpyrrolidone) (PVP), and (E) Tris HCl (TRIS) in the non-stressed and stressed samples; TRH = the sample stressed with high temperature and high humidity, UV/Vis = the sample stressed with UV/Vis light; Table S1: FT-IR/ATR and NIR vibrational bands of the examined drugs and excipients; Table S2: Effect of high temperature/high humidity (70 °C/80% RH) and UV/Vis light (94.510 kJ/m^2^) on excipients, HEC, MAN, PVA, PVP, and TRIS.
