# Supplementary material for: Spectroscopic and Chemometric Evaluation of the Stability of Timolol, Naphazoline, and Diflunisal in the Presence of Reactive Excipients Under Forced Degradation Conditions
Source: Molecules. 2025 Sep 19;30(18):3807. doi: 10.3390/molecules30183807 (PMC12473119; doi:10.3390/molecules30183807)
Supplement: Supplementary file 1 [file molecules-30-03807-s001.zip › molecules-3834139-supplementary.pdf]

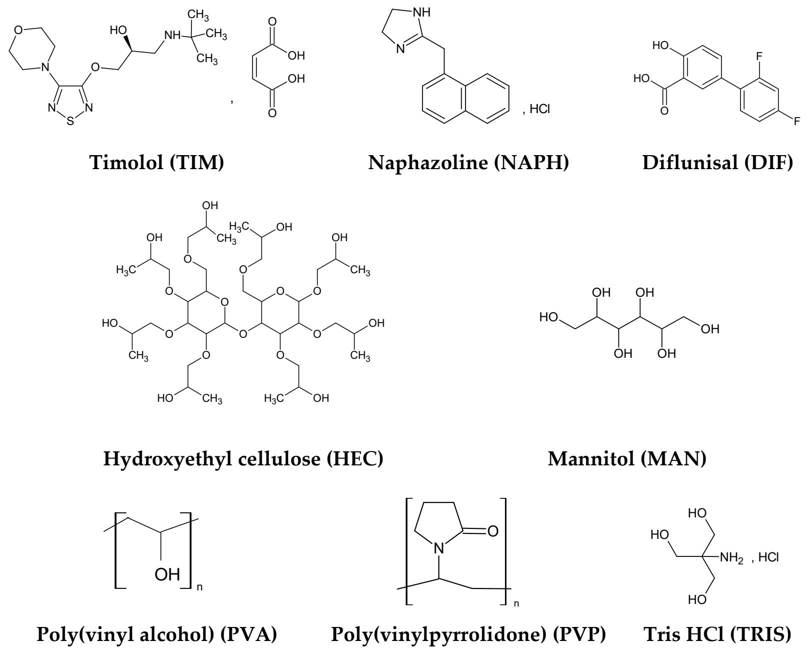

Figure S1. The chemical structures of the drugs and excipients being tested.

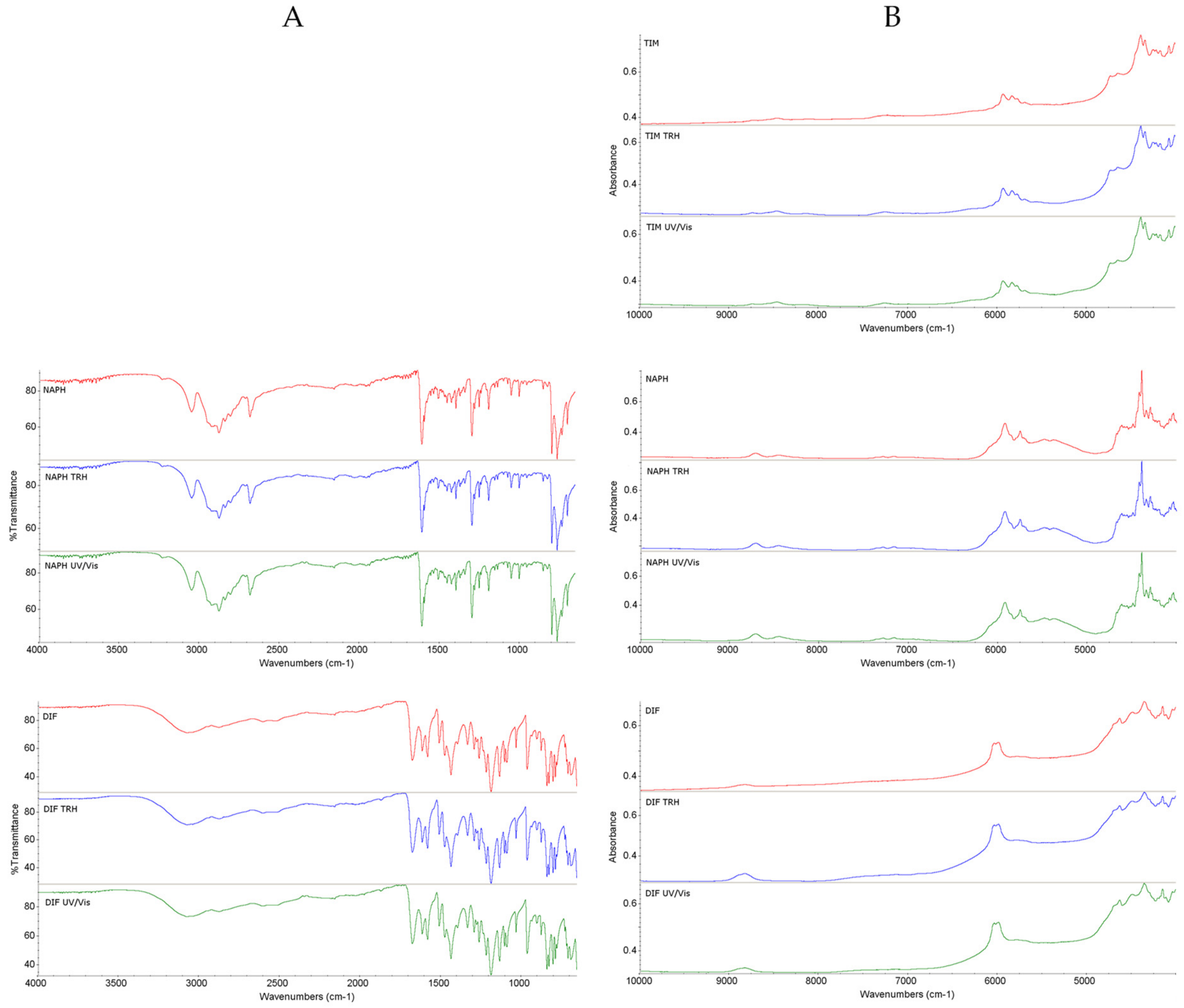

Figure S2. The spectra of the non-stressed and stressed samples of the drugs: (A) FT-IR spectra of NAPH and DIF; (B) NIR spectra of TIM, NAPH and DIF; TRH = the sample stressed with high temperature and high humidity, UV/Vis = the sample stressed with UV/Vis light.

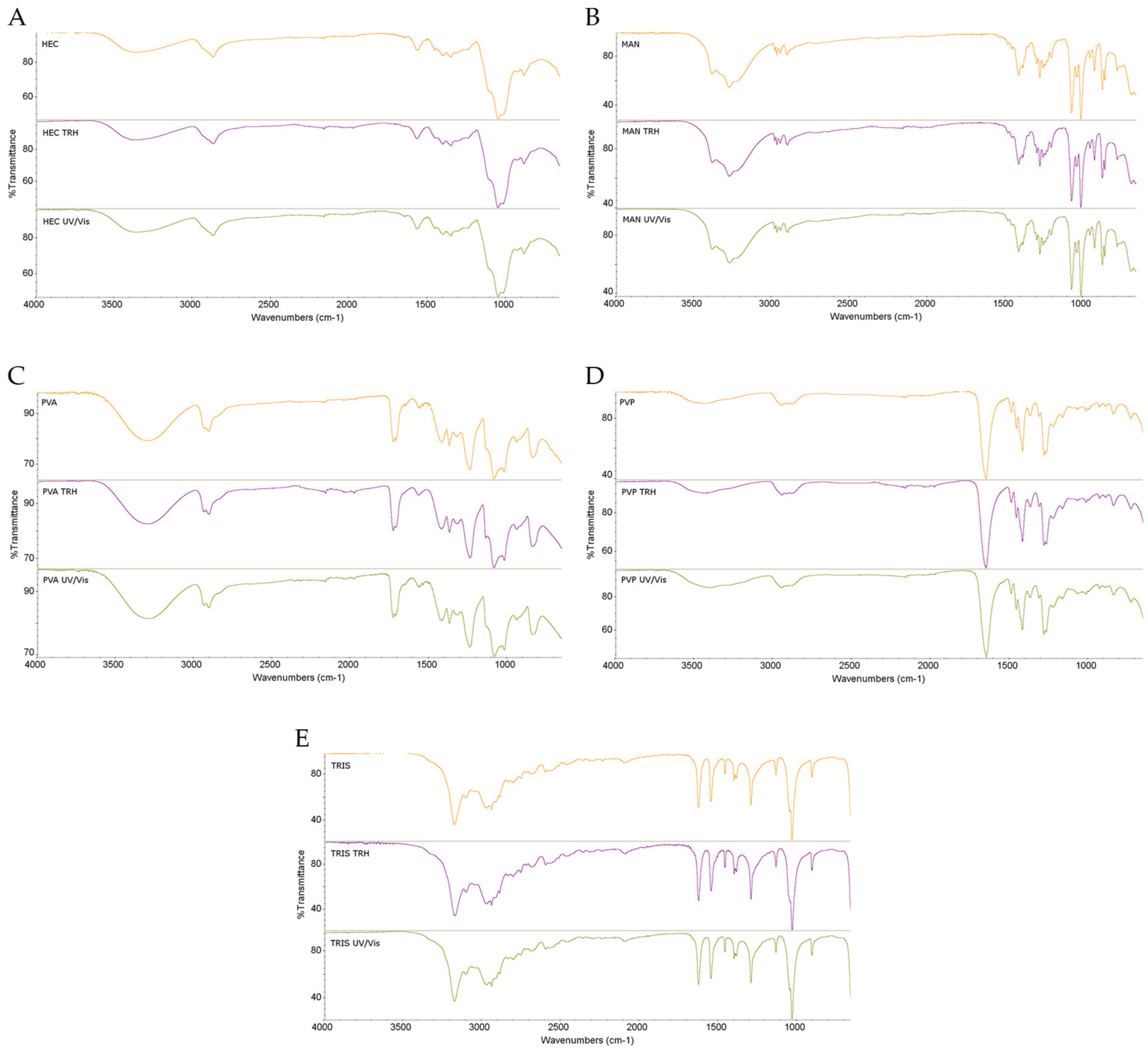

Figure S3. FT-IR/ATR spectra of: (A) HEC, (B) MAN, (C) PVA, (D) PVP and (E) TRIS in the non-stressed and stressed samples; TRH = the sample stressed with high temperature and high humidity, UV/Vis = the sample stressed with UV/Vis light.

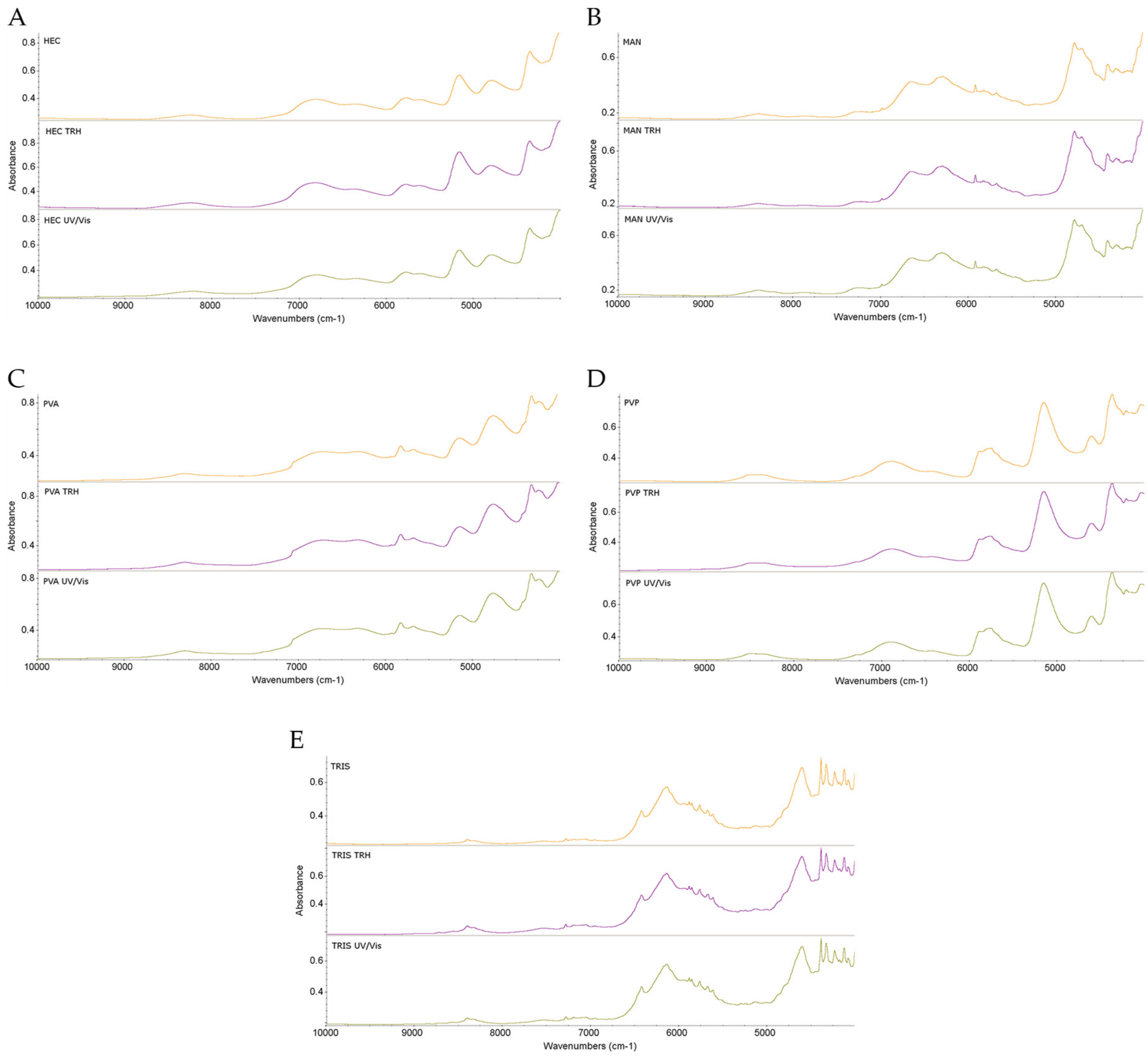

Figure S4. NIR spectra of: (A) HEC, (B) MAN, (C) PVA, (D) PVP and (E) TRIS in the non-stressed and stressed samples; TRH = the sample stressed with high temperature and high humidity, UV/Vis = the sample stressed with UV/Vis light.

Table S1. FT-IR/ATR and NIR vibrational bands of the examined drugs and excipients

Compound	FT-IR/ATR bands [cm^{-1}]	Vibrations	NIR bands [cm^{-1}]	Vibrations
TIM	3300	O–H stretching	8751, 8460	CH_3, CH_2 second overtones
	3088	N–H	5984, 5845, 5697	CH_3, CH_2 first overtones
	2966, 2890	C–H in -CH_3 stretching	5315	C=O second overtones (maleate)
	2260–2210	C=N	4827, 4727	O–H combinations
			4662	N–H combinations
	1708	C=O (maleate)	4360, 4272	CH_3 combinations
	1685, 1670	C–N stretching in aliphatic amine	4041	C–H and C–C combinations
	1574	C=C		
	1489, 1450	C–C stretching		
	1228, 1210	O–H bending		
	1078, 1014	C–N stretching in aliphatic amine		

NAPH	3217	N–H stretching		
	3054, 2948, 2912	C–H stretching in aromatic ring	8728, 8478	CH_2, C–H second overtones
	2884, 2842	C–H in CH_2 stretching	7327, 7183	C–H first overtone combinations
	1623	N–H stretching	6158, 5929	C–H in aromatic ring first overtones
	1404	C–C in five-membered ring stretching	4619, 4568	N–H combinations
	1280	C–N stretching	4568	C–H and C=O combinations
	1056, 1004	C–H in plane bending	4494, 4393	CH_2 and C–**H** combinations
	801	C–H out of plane bending	4244, 4184, 4152, 4045	CH_2, C–H second overtones
DIF	3077–2986	C–H stretching in aromatic ring	8835	CH_2, C–H second overtones
	2879	O–H stretching in COOH	7058	C–OH first overtones
	2618–2532	O–H stretching in intramolecular H-bonded o-substituted acid	6049, 5988	C–H in aromatic ring first overtones
	1876, 1845	C–H out of plane deformation	5225	C=O overtones in COOH
			4647, 4522	C–H and C=O combinations
	1685, 1623	C=O stretching in COOH	4393	C–H combinations
	1582, 1472	C–C stretching in aromatic ring	4166	CH_2, C–H second overtones
	1410–1376	C–OH bending in OH associated with C=O through H-bonding		
	1329–1298	O–H deformations		
	1207–1095	C=C stretching		
	1190	C–O stretching in aromatic ring		
HEC	3400, 3372	O–H stretching	8437, 8261	CH_2, C–H second overtones
	3000–2750	C–H stretching	6873, 6664	C–H first overtones combinations
	1566, 1406	C–H bending	6359	C–H first overtones
	1063, 1050	C–O–C stretching	5767, 5628	C–H first overtones
	892, 887	C–O in glycosidic bond	5239, 4814	O–H combinations
			4457	C–H combinations
MAN	3400, 3382	O–H stretching	8437	CH_2, C–H second overtones
	3276, 3200	O–H stretching	6664	O–H first overtones
	2969, 2948	C–H bending	6313, 5929, 5836	C–H first overtones
	1450, 1432	C–H bending	4795, 4712	O–H combinations
	1417	C–H in CH_2 deformation	4416, 4318	C–H combinations
	1291, 1282, 1277	O–H deformation		
	1081, 1076	C–O stretching		
	1016	C–O stretching		
PVA	3305, 3275	O–H stretching	8321	CH_2, CH second overtones
	2925–2800	C–H stretching in alkans	6771	O–H first overtones
	1725, 1719	C–O stretching	6317, 5943	C–H first overtones
	1424, 1413	C–H bending	5156, 4777	O–H combinations
	1363, 1325	C–H	4430, 4249	C–H combinations
	1251	C–O stretching		
	1085, 1039	C–O stretching		

PVP	2951, 2946	C–H stretching	8515, 8349	CH_2, C–H second overtones
	1654, 1646	C=O stretching	7303, 6951	C–H first overtone combinations
	1424, 1370	CH2 bending	6419, 5952	C–H first overtones
	1287	C–N stretching	5840	C=O overtones
			4666	N–H combinations
			4422, 4381	C-H combinations
TRIS	3351	O–H stretching	8737, 8445, 8409	CH_2, C–H second overtones
	3195, 3178	N–H stretching	7590, 7210	CH first overtone combinations
	2979, 2938	C–H stretching	6535, 6453, 6155	C–H first overtones
	1626, 1589	N–H bending	5684, 5619	CH_2 first overtones
			4650, 4619	N–H combinations
	1462, 1485	C–H bending	4244	CH_2, C–H combinations
	1292	C–N stretching	4138	CH_2, C–H second overtones
	1215	C–C stretching		
	1034	C–O stretching		

Table S2. Effect of high temperature/high humidity (70°C/80% RH) and UV/Vis light (94.510 kJ/m2) on excipients, HEC, MAN, PVA, PVP, and TRIS

**Excipients**	**IR spectra**	**High temperature/high humidity**		**UV/Vis light**	
		**PCA**	**HCA**	**PCA**	**HCA**
**HEC**	FT-IR	–	–	+	+
	NIR	+	+++	++	++
**MAN**	FT-IR	–	+	–	+
	NIR	+	++	–	+
**PVA**	FT-IR	+	+	–	+
	NIR	–	+	–	++
**PVP**	FT-IR	+	+	++	++
	NIR	++	++	+	+
**TRIS**	FT-IR	+	+	+	+
	NIR	+	++	+	++

The influence of stress conditions: +++ strong, ++ medium, + week, – no impact.
